# Initial Transarterial Chemoembolization (TACE) Using HepaSpheres 20–40 µm and Subsequent Lipiodol TACE in Patients with Hepatocellular Carcinoma > 5 cm

**DOI:** 10.3390/life11040358

**Published:** 2021-04-18

**Authors:** Su Min Cho, Hee Ho Chu, Jong Woo Kim, Jin Hyung Kim, Dong Il Gwon

**Affiliations:** Asan Medical Center, Department of Radiology, University of Ulsan College of Medicine, 88 Olympic-ro 43-gil, Songpa-gu, Seoul 05505, Korea; sumin943@gmail.com (S.M.C.); chuzzang1224@gmail.com (H.H.C.); ewooya@empas.com (J.W.K.); jhkimrad@amc.seoul.kr (J.H.K.)

**Keywords:** DEB-TACE (drug-eluting bead transarterial chemoembolization), hepatocellular carcinoma, Cis-TACE (cisplatin-based lipiodol transarterial chemoembolization)

## Abstract

Purpose: To investigate clinical outcomes of drug-eluting bead transarterial chemoembolization (DEB-TACE) using HepaSpheres 20–40 µm in diameter and subsequent cisplatin-based lipiodol TACE (Cis-TACE) in patients with hepatocellular carcinoma (HCC) > 5 cm. Materials and Methods: This study included 39 consecutive patients (34 men, 5 women; mean age, 63.5 years; range, 39–80 years) who underwent DEB-TACE using HepaSpheres 20–40 µm as first-line treatment for HCC > 5 cm (mean diameter, 8.2 cm; range, 5.1–13 cm) between September 2018 and August 2019. Patients with new tumors, residual tumors, or tumor growth after initial DEB-TACE underwent subsequent Cis-TACE. Results: All 39 patients underwent initial DEB-TACE successfully, with 35 (89.7%) and three (7.7%) patients experiencing minor and major complications, respectively. After initial DEB-TACE, one patient (2.6%) achieved complete response (CR), 35 (89.7%) achieved partial response (PR), and three (7.7%) experienced progressive disease (PD). During a median follow-up period of 14.4 months (range, 0.6–23 months), 23 patients underwent Cis-TACE, with 11, three, and nine achieving CR, PR, and PD, respectively. The median overall survival time was 20.9 months (95% confidence interval (CI), 18.6–23.2 months), the median time to progression was 8.8 months (95% CI, 6.5–11.1 months), and the median time to local tumor recurrence was 16 months (95% CI, 7.4–24.6 months). Conclusions: DEB-TACE using HepaSpheres 20–40 µm in diameter can be a safe and effective initial treatment method in patients with HCC > 5 cm. Subsequent Cis-TACE constitutes a good adjuvant method to enhance tumor response after initial DEB-TACE.

## 1. Introduction

Although conventional transarterial chemoembolization (C-TACE) using emulsions of lipiodol and chemotherapeutic agent(s) has been found to improve survival rate in patients with unresectable hepatocellular carcinoma (HCC) [1,2], this method has important drawbacks associated with techniques and scheduling, which have not yet been standardized [3]. Drug-eluting bead TACE (DEB-TACE) has several advantages over C-TACE, such as the delivery of higher concentrations of chemotherapeutic agents directly to tumors, lower rates of systemic complications, greater efficacy in advanced stage or large tumors, and better standardization of the procedure itself [4,5,6,7,8,9,10].

Selecting the optimal therapeutic approach for patients with large, inoperable HCCs is important. The benefits of DEB-TACE over C-TACE are presently unclear, with some studies reporting significantly better patient outcomes with DEB-TACE [5,10,11], while others show no differences [12,13,14]. Tumor size may not be an indicator of efficacy and may not be a criterion for choosing between C-TACE and DEB-TACE [15]. However, a recent study reported that patients with large (>5 cm) HCC may be more likely to show an objective tumor response with DEB-TACE than with C-TACE [10].

No consensus has been reached on the optimal size of microspheres for DEB-TACE treatment. The use of smaller, non-absorbable drug-eluting microspheres showed greater distal penetration and more effective embolization than larger microspheres [16,17,18,19,20,21]. Furthermore, complication rates were not increased by using smaller (100–300 µm) microspheres [17,18]. Studies have evaluated the safety and efficacy of DEB-TACE using HepaSpheres 30–60 μm in diameter and loaded with doxorubicin [10,22,23,24]. The objective tumor regression rate in patients with HCCs > 5 cm using HepaSpheres 30–60 µm in diameter was 81.3% [10], much higher than the rate of 32% obtained using larger HepaSpheres (50–100 µm) [4].

HepaSpheres 20–40 µm in diameter (Merit Medical, South Jordan, UT, USA) are a new size of loadable microspheres. To date, however, no clinical data have been published regarding the results of DEB-TACE using HepaSpheres 20–40 µm. Thus, the aim of this study was to investigate clinical outcomes of DEB-TACE using HepaSpheres 20–40 µm in diameter and subsequent Cis-TACE in patients with HCC > 5 cm.

## 2. Materials and Methods

### 2.1. Patient Population

This study was approved by the institutional review board, which waived the need for written informed consent because of the retrospective nature of this study. This study enrolled 39 consecutive patients (34 men, 5 women; mean age, 63.5 years; range, 39–80 years) who underwent DEB-TACE using HepaSpheres 20–40 µm as first-line treatment for HCCs > 5 cm between September 2018 and August 2019. Patients who had previously undergone surgical resection, ablation therapy, or TACE were excluded because previous treatments may influence the effectiveness of DEB-TACE. Indications for DEB-TACE in our institution are (i) HCCs not considered eligible for ablation therapies (i.e., radiofrequency ablation or percutaneous ethanol injection) because of tumor size > 5 cm, multiple lesions (i.e., more than three), vascular invasion, or technical contraindications (i.e., subcapsular location or tumor adjacency to hepatic vasculature, main bile ducts, or intestinal loops); and (ii) HCCs regarded as unresectable because of either advanced stage or insufficient hepatic reserve. Advanced liver disease (Child–Pugh class C), portal vein tumor thrombosis, and extrahepatic metastasis are not considered contraindications to DEB-TACE in our institution. Patients with HCC were considered ineligible for treatment with DEB-TACE for HCC if they had any contraindication to an arterial procedure, such as impaired clotting (platelet count < 50,000/mm^3^ or prothrombin activity < 50%), bacterial infection, or renal failure (i.e., estimated glomerular filtration rate < 30 mL/min/1.73 m^2^). The baseline characteristics of the 39 included patients are summarized in Table 1.

### 2.2. Preparation for HepaSphere Treatment

Each vial of HepaSphere 20–40 µm was loaded with 50 mg of doxorubicin according to the procedure recommended by the manufacturer. Briefly, 10 cc of a solution of doxorubicin was added to the vial containing HepaSpheres and agitated for 10 min, followed by the addition of another 10 cc of doxorubicin solution. The vial was agitated periodically for 1 h to complete the ionic bonding of the doxorubicin [10,22]. The supernatant was removed from the vial, and nonionic contrast medium was added to the supernatant to obtain a final injectable volume of 30 cc.

### 2.3. HepaSphere and Cisplatin-Based Lipiodol Transarterial Chemoembolization

All TACE procedures were performed by one of three interventional radiologists (with 3, 12, and 18 years of experience, respectively). Following puncture and cannulation of the right femoral artery, superior mesenteric and common hepatic arteriographies were performed with a 5-F catheter (Rősch hepatic catheter, Cook, Bloomington, IN, USA) to assess vascular anatomy, tumor location and extent, and the patency of portal flow. After selective or super-selective catheterization of the feeding artery using a microcatheter (diameter; 1.9, 2.0, or 2.2 Fr), 50–100 µm of nitroglycerin was injected through the microcatheter into the target artery to dilate the tumor-feeding artery and to prevent proximal embolization [10,22]. The HepaSphere suspension was slowly injected at a rate of 1–3 mL/min until near stasis was achieved, followed by a wait for 5 min to allow the microspheres to redistribute within the lesion and to be pushed more distally by the blood flow. After the waiting period, more microspheres were injected, if necessary [10,22].

After DEB-TACE of the hepatic artery, angiographies of extrahepatic collateral vessels were performed during the same session, based on tumor location, especially in patients with no or insufficient tumor blush on hepatic angiograms. If tumor blush was visible in the collateral vessels, DEB-TACE of the vessels was performed. The HepaSphere suspension was infused super-selectively into each feeding branch, followed by embolization of the collateral vessels using a 1:10 mixture of n-butylcyanoacrylate with lipiodol.

Patients with partial tumor response during follow-up were subjected to cisplatin-based lipiodol TACE (Cis-TACE). Briefly, TACE was performed by infusing 2 mg/kg body weight of cisplatin (Cisplan; Donga-ST, Seoul, Korea) into the target segmental or subsegmental artery for 15 min using a microcatheter. Some cisplatin was mixed 1:1 with iodized oil to form an emulsion (lipiodol, Guerbet, Roissy, France; 2–15 mL), which was infused into the subsegmental or more peripheral level feeding artery, followed by embolization with gelfoam slurry (Upjohn, Kalamazoo, MI, USA) until arterial flow stasis was achieved [2,10].

### 2.4. Follow-Up

Following DEB-TACE, patients were closely monitored in a ward for at least 3 days to detect and manage any adverse events or post-embolization syndrome. Patients were discharged when there was no discomfort or after improvement of complications. Discharge was delayed if patients experienced any adverse events requiring major medical attention or therapy. Patients were followed-up routinely by physical examination and laboratory tests (blood count, α-fetoprotein, and liver function tests) at 1 month intervals. One to three months after DEB-TACE, depending on patient circumstances, patients were followed-up by dynamic contrast-enhanced abdominal computed tomography (CT) or magnetic resonance imaging (MRI), with subsequent management plans determined by multidisciplinary teams, depending on each patient’s general condition, laboratory findings, and evaluation of tumor response. Patients underwent subsequent follow-up CT or MRI every 2–3 months until recurrence of HCC. Subsequent Cis-TACE was performed following the detection of residual tumors, new tumors, or tumor growth. Patients were followed-up until March 2020.

### 2.5. Definitions and Statistical Analysis

Radiologic response was defined as complete response (CR), partial response (PR), stable disease (SD), or progressive disease (PD) [19]. Time to progression (TTP) was measured from the date of DEB-TACE to the date of follow-up imaging at which PD was first observed. Time to local tumor recurrence (TTL) was also measured from the date of DEB-TACE to the date of the follow-up CT or MRI at which viable tumor was first observed around the treated HCC.

Complications were classified as major or minor, according to the guidelines of the Society of Interventional Radiology (SIR) Standard of Practice Committee [25]. Major complications were defined as events requiring additional treatment, including a hospital stay beyond observation status, increased level of care, or causing substantial morbidity or death (SIR classifications C–F). All other complications were classified as minor (SIR classifications A and B).

Continuous variables are presented as means and standard deviations, and categorical variables as absolute numbers and percentages. Normally distributed variables were compared using paired t tests, and non-normally distributed variables were compared using Wilcoxon signed-rank tests. Cumulative TTP and TTL were determined using the Kaplan–Meier method. All statistical analyses were performed using IBM SPSS Statistics for Windows, version 23.0 (IBM Corp., Armonk, NY, USA).

## 3. Results

### 3.1. Technical Outcomes after DEB-TACE Using HepaSpheres 20–40 µm

All 39 patients underwent the initial DEB-TACE procedure successfully. The mean number of vials injected into each patient was 2.2 (range, 1–3) and the mean dose of doxorubicin was 110 mg (range, 50–150 mg). Of the 39 patients, 38 achieved stasis after the injection of HepaSphere suspension was completed and did not require additional embolic material, whereas one patient required administration of additional embolic material (Embosphere 100–300 µm, Merit Medical, South Jordan, UT, USA) to achieve stasis of the feeding artery. A parasitic supply from the right inferior phrenic artery (IPA) was noted in 17 patients.

### 3.2. Safety

Of the 39 patients, 35 (89.7%) experienced minor complications after DEB-TACE, including fever, abdominal pain, and nausea. All of these patients were treated with intravenous analgesia with/without antibiotics and were discharged after symptoms improved. In one patient, asymptomatic small bilomas were detected on the 1-month follow-up CT.

Three patients (7.7%) experienced major complications associated with DEB-TACE. All three patients experienced abscesses in necrotic tumors, and one had multiple bilomas. In one patient, an abscess occurred immediately after DEB-TACE; this patient died 18 days later due to acute respiratory distress syndrome that occurred while being treated with the abscess. A second patient was discharged without specific findings other than fever, but he was re-admitted through the emergency room 20 days later due to abdominal pain, with diagnostic imaging confirming an abscess. Hepatic failure occurred during hospitalization and he died due to hepatic failure 2 months after the DEB-TACE procedure. The abscess in the third patient occurred immediately after the DEB-TACE procedure; this patient underwent percutaneous catheter drainage and was discharged without significant sequelae 52 days after the procedure.

### 3.3. Tumor Response

**Figure 1:**
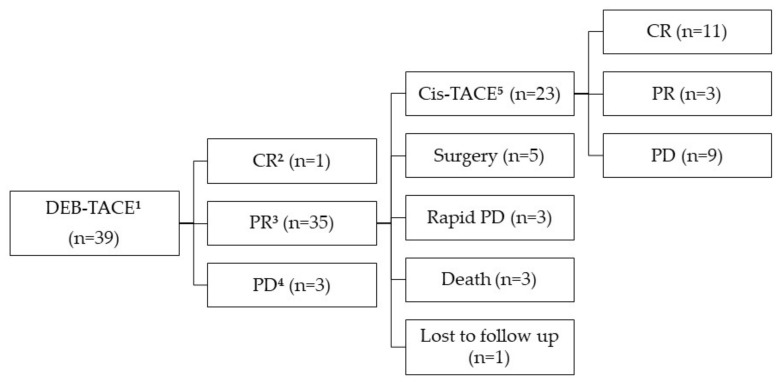
Flow chart of initial tumor response after DEB-TACE and follow-up outcomes. DEB-TACE ^1^ = drug-eluting bead transarterial chemoembolization, CR ^2^ = complete response, PR ^3^ = partial response, PD ^4^ = progressive disease, Cis-TACE ^5^ = cisplatin-based lipiodol transarterial chemoembolization.

Figure 1 shows a flow chart of the subsequent treatment of the 39 patients who underwent DEB-TACE. Of these patients, one (2.6%) achieved CR, 35 (89.7%) achieved PR, and three (7.7%) achieved PD, making the objective response (OR) rate 92.3%. Ten patients who achieved PR after DEB-TACE did not undergo subsequent planned Cis-TACE because of rapid tumor progression (n = 6), early death due to infected tumor necrosis (n = 2), gastric variceal bleeding (n = 1), and loss to follow-up (n = 1). Five patients underwent a hemihepatectomy at a mean of 44 days (range, 28–63 days) after DEB-TACE. The remaining 23 patients who achieved PR after DEB-TACE underwent subsequent Cis-TACE. Of these patients, 11 (47.8%) achieved CR, three (13.1%) achieved PR, and nine (39.1%) achieved PD.

**Figure 2:**
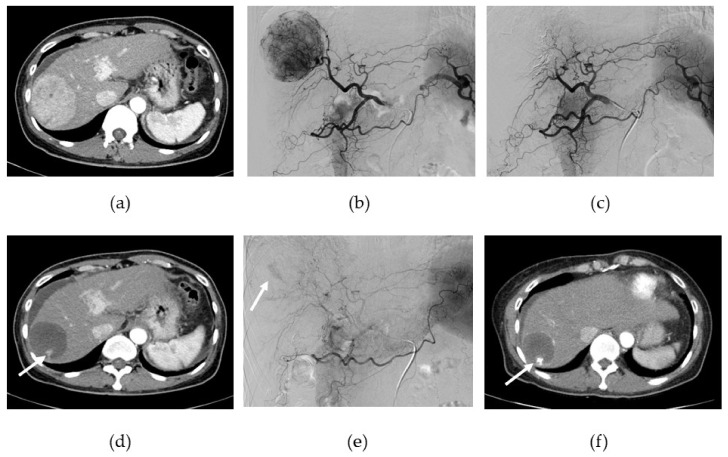
A 53-year-old woman who underwent DEB-TACE and one subsequent session of Cis-TACE for a single HCC 7.2 cm in size. (**a**) Contrast-enhanced axial computed tomography (CT) image in the arterial phase before initial DEB-TACE, showing an arterial enhancing mass in the right hemiliver. (**b**) Common hepatic arteriography of the patient during initial DEB-TACE, showing a hypervascular tumor in the right hemiliver. (**c**) Angiogram after selective embolization of the tumor-feeding arteries with HepaSpheres, showing complete devascularization of the tumor in the right hemiliver. (**d**) Enhanced axial CT image 5 weeks after DEB-TACE, showing PR with small residual arterial enhancement (arrow). (**e**) Common hepatic arteriography during subsequent Cis-TACE, showing a small enhancing lesion (arrow) in the center of the necrotic tumor. The tumor-feeding arteries were selectively embolized (not shown). (**f**) Enhanced axial CT image 4 weeks after subsequent Cis-TACE, showing no demonstrably enhancing portion in the liver and treated tumor, with lipiodol accumulating solely in the viable portion (arrow).

During the median follow-up period of 14.4 months (range, 0.6–23 months), 23 patients underwent at least one subsequent Cis-TACE session, with six, six, four, four, and three patients undergoing one, two, three, four, and five procedures, respectively. Hepatic angiography after the first Cis-TACE procedure showed hepatic arterial damage (HAD) (stenosis) in two of these patients, with most of the viable HCCs supplied by fine feeders from the segmental and/or subsegmental arteries. Of these 23 patients, 11 achieved CR, including six, two, two, and one who achieved CR after one, two, three, and four subsequent Cis-TACE procedures, respectively (mean, 1.8 sessions) (Figure 2). Three patients achieved PR and nine had PD.

### 3.4. Survival and Follow-Up Outcomes

**Figure 3:**
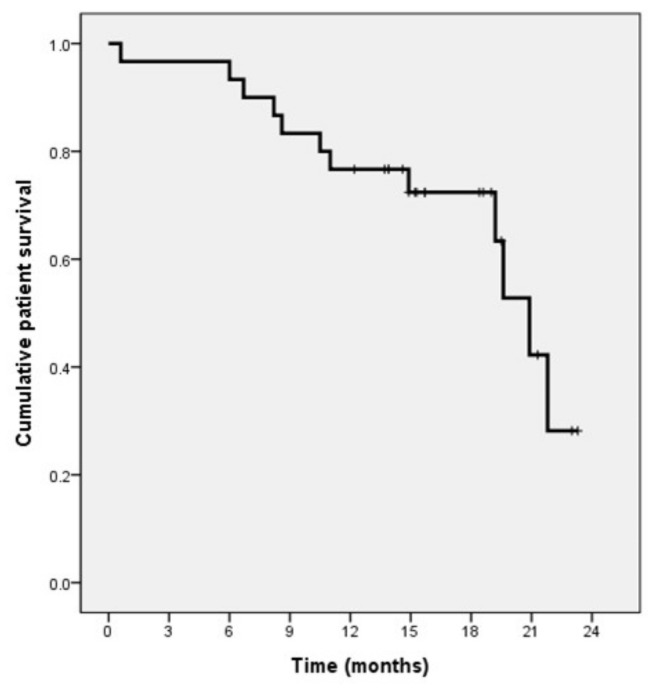
Kaplan–Meier analysis of overall survival in patients with HCCs > 5 cm who underwent DEB-TACE.

**Figure 4:**
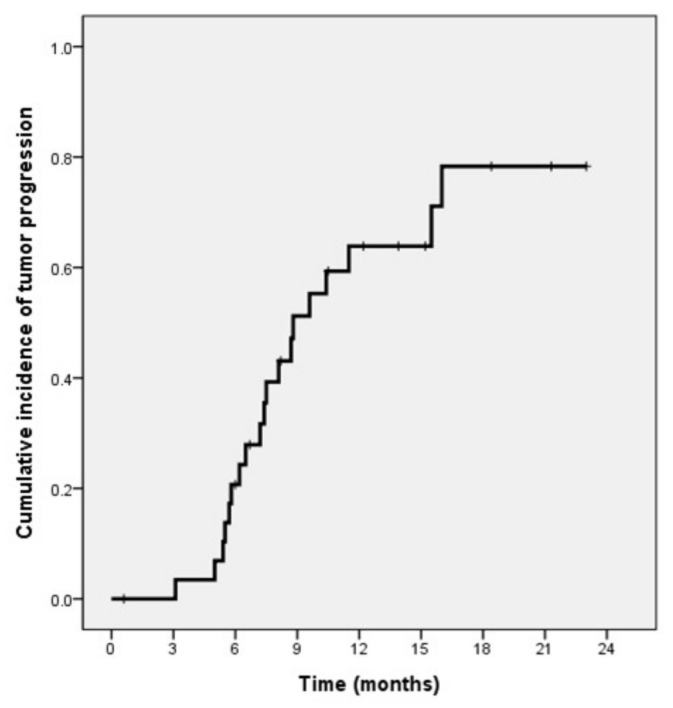
Kaplan–Meier analysis of time to progression in patients with HCCs > 5 cm who underwent DEB-TACE.

Of the 39 patients, one was lost to follow-up, with clinical follow-up information until death or the end of the study (31 August 2020) available for the other 38 patients. Of these 38 patients, 16 died during follow-up, whereas 22 remained alive. The median patient survival time was 20.9 months (95% confidence interval (CI), 18.6–23.2 months), and the cumulative survival time at 1 year was 74% (Figure 3). The median TTP was 8.8 months (95% CI, 6.5–11.1 months), and the TTP at 1 year was 36% (Figure 4). The median TTL was 16 months (95% CI, 7.4–24.6 months), and the TTL at 1 year was 57%.

## 4. Discussion

The present study showed that DEB-TACE with a new size of HepaSpheres, 20–40 µm in diameter, was technically feasible and safe in all 39 patients with HCCs > 5 cm. Moreover, these HepaSpheres were an effective embolic agent achieving an OR rate of 92.3% (36/39), including a CR rate of 2.6% and a PR rate of 89.7%. This OR rate was similar to or slightly higher than the OR rates of 75–100% observed in patients with HCCs > 5 cm using HepaSpheres 30–60 µm in diameter [4,24]. Smaller-caliber microspheres with an increased surface area may be capable of greater distal penetration and delivery of higher concentrations of chemoembolization agents [16,17,18,19,20,21]. Thus, DEB-TACE with smaller-caliber microspheres should result in a better tumor response and higher survival rate than DEB-TACE with larger-caliber microspheres [18,26].

Tumor response and local tumor recurrence after DEB-TACE were found to be significantly associated with large (>5 cm) tumor size [4,24,27,28]. Because additional treatment in patients with HCCs > 5 cm should result in a higher response rate during the follow-up period, subsequent Cis-TACE could be useful in treating these large HCCs, because most of the viable tumor after initial DEB-TACE is usually supplied by small feeding arteries or injured feeders. Possible risk factors for HAD include dose of doxorubicin and number of TACE sessions, although evidence is lacking regarding the relatively better prognosis in patients with and without HAD. The incidence of HAD was reported to be higher after DEB-TACE than after C-TACE because the dose of doxorubicin was higher in the DEB-TACE group [29]. In addition, a greater number of TACE sessions was found to increase the incidence of HAD, with this incidence being 24% after three or more sessions of TACE [30]. In the present study, the incidence of HAD was reduced and the embolization of the residual tumor increased by performing Cis-TACE after initial DEB-TACE. Of the 23 patients who underwent subsequent Cis-TACE, two presented with HAD (stenosis) after initial DEB-TACE. Eleven (47.8%) of the 23 patients who underwent Cis-TACE after DEB-TACE achieved CR after a mean of 1.8 sessions (range, 1–4 sessions) of Cis-TACE. The present study also found that the median TTP was 8.8 months and the median TTL was 16 months, further suggesting that Cis-TACE after DEB-TACE in patients with large (>5 cm) HCC would be a good adjuvant method to achieve a better tumor response.

In previous studies with drug-eluting microspheres, multivariate analysis showed that overall survival was shorter in patients having larger and multiple lesions, as well as tumors with ill-defined infiltrative borders [24]. In the present study, the median survival time was 20.9 months and the 1-year cumulative survival rate was 74%. The latter rate was similar to the rates of 60–74% observed in previous studies of TACE in patients with HCCs > 5 cm [31,32,33].

Recently, studies on the clinical outcome of C-TACE and transarterial radioembolization (TARE) for the intermediate stage have been actively conducted. Several studies reported that C-TACE and TARE showed similar results in safety and efficacy in intermediate stage HCC treatment [34,35,36]. According to the results of previous studies, median overall survival after C-TACE was up to 6–17.4 months, and median overall survival after TARE was reported to be 6–20.5 months [34]. The median survival in this study using DEB-TACE was 20.9 months, which is similar to those of C-TACE and TARE for unresectable HCC. However, it is difficult to compare the median patient survival of this study with results of C-TACE and TARE studies, as all of the study patients had large HCC > 5 cm.

In the present study, 89.7% of patients experienced post-embolization syndrome, which may be associated with the large area of chemoembolization due to large HCC. Four patients experienced major complications associated with DEB-TACE, including three with abscess in the necrotic tumor and one with biloma. Biliary complications after DEB-TACE are thought to be due to ischemic injury of the peribiliary plexus resulting from local doxorubicin toxicity. Although treatment with small microspheres has been associated with increased intensity of biliary complications [16,17,18,19,20,21], the present study found that the rate of all types of biliary complications was 5.1%, similar to those previously reported (0–5.7%) for DEB-TACE using small microspheres (40–100 µm) [37,38,39].

This study had several limitations. First, it was a retrospective single-center study with a relatively small patient population. However, to our knowledge, this study was the first to evaluate the safety and efficacy of DEB-TACE using HepaSpheres of diameter 20–40 µm in patients with HCCs > 5 cm. Second, chemoembolization techniques varied among these patients. However, this possible bias was minimized by all patients being treated at a single-center with an internal standard procedure.

## 5. Conclusions

In conclusion, DEB-TACE using HepaSpheres 20–40 µm in diameter can be a safe and effective initial treatment method in patients with HCCs > 5 cm. Subsequent Cis-TACE constitutes a good adjuvant method to enhance tumor response after initial DEB-TACE.

## Figures and Tables

**Table 1:** Baseline characteristics of the 39 patients with HCCs > 5 cm who underwent DEB-TACE.

Characteristics	Value
Age (y)	63.8 ± 11.5
Sex	
Male	34 (87.2)
Female	5 (12.8)
Etiology of cirrhosis	
Hepatitis B virus infection	26 (66.7)
Hepatitis C virus infection	2 (5.1)
Alcohol	4 (10.3)
Others	7 (17.9)
Child–Pugh classification	
A	31 (79.5)
B	8 (20.5)
BCLC ^1^ classification	
A	11 (28.2)
B	14 (35.9)
C	14 (35.9)
Initial distant metastasis	3 (7.7)
Initial lymph node metastasis	4 (10.3)
Maximum tumor size	
Mean (cm)	8.2 ± 2.4
Range (cm)	5.1–13
5–10 cm	27 (69.2)
10 cm	12 (30.8)

Results are reported as mean ± standard deviation or number (%). Continuous data are expressed as mean standard deviation, and categorical data are expressed as number (%), otherwise unspecified. BCLC ^1^ = Barcelona clinic liver cancer.
